# Molecular cloning and expression of key gene encoding hypothetical DNA polymerase from *B. mori* parvo-like virus

**DOI:** 10.1590/S1415-47572010005000083

**Published:** 2010-12-01

**Authors:** Junhong Zhang, Guohui Li, Huiqing Chen, Xiaogang Li, Meng Lv, Keping Chen, Qin Yao

**Affiliations:** Institute of Life Sciences, Jiangsu University, ZhenjiangChina

**Keywords:** *Bombyx mori parvo-like virus*, DNA polymerase, recombinant protein, Sf-9

## Abstract

*Bm*PLV-Z is the abbreviation for *Bombyx mori* parvo-like virus (China isolate). This is a novel virus with two single-stranded linear DNA molecules, viz., VD1 (6543 bp) and VD2 (6022 bp), which are encapsidated respectively into separate virions. Analysis of the deduced amino acid sequence of VD1-ORF4 indicated the existence of a putative DNA-polymerase with exonuclease activity, possibly involved in the replication of *Bm*PLV-Z. In the present study, a recombinant baculovirus was constructed to express the full length of the protein encoded by the *VD1-ORF4* gene (3318 bp). In addition, a 2163-bp fragment amplified from the very same gene was cloned into prokaryotic expression vector pET-30a and expressed in *E.coli* Rosetta 2 (DE3) pLysS. The expressed fusion protein was employed to immunize New Zealand white rabbits for the production of an antiserum, afterwards used for examining the expression of the protein encoded by *VD1-ORF4* gene in Sf-9 cells infected with recombinant baculovirus. Western blot analysis of extracts from thus cells infected revealed a specific band of about 120 kDa, thereby indicating that the full length protein encoded by the *VD1-ORF4* gene had been successfully and stably expressed in Sf-9 cells.

## Introduction

Parvoviruses are small icosahedral, non-enveloped particles 18-26 nm in diameter, which possess a small linear single-stranded DNA in the genome characterized by inverted terminal repeats (ITRs) and palindromic sequences folding to form a hairpin structure. It is generally thought that DNA synthesis of parvoviruses is initiated by a self-priming mechanism (Tattersall *et al.*, 1973; Astell *et al.*, 1985; Bando *et al.*, 1990). Unlike parvoviruses, the genome of *Bm*PLV-Z is composed of two separated linear single-stranded DNA molecules (VD1 and VD2), each of which is encapsidated separately in virions. The genome was sequenced and published in 2005 (Accession Number DQ017268 and DQ017269, in order to characterize its structure. Sequence analysis showed VD1 consist of 6,543 nt including inverted terminal repeats (ITRs) of 224 nt, and VD2 of 6,022 nt with like repeats of 524 nt. A 53 bp consensus sequence was found at both the VD1 and VD2 terminal. Unlike other parvoviruses and densovirus, the ITRs of *Bm*PLV-Z, besides being imperfect, were incapable of forming palindrome structures. Furthermore, whereas VD1 genome contains four ORFs, of which ORF4 located in the genomic right-hand portion encodes a hypothetical DNA polymerase, and VD2 genome contains two major ORFs (Wang *et al.*, 2007; Li *et al.*, 2009). The unique properties of *Bm*PLV-Z imply a possible different replication mechanism to that of densoviruses or vertebrate parvovirus (Li *et al.*, 2009; Tijssen *et al.*, 1995). In fact, based on the analysis of deduced amino acid sequences (Tijssen *et al.*, 1995; Kapitonov *et al.*, 2006), it was concluded that there is a DNA polymerase-like protein encoded by *VD1-ORF4* in *Bm*PLV-Z, which is not found in densoviruses or vertebrate parvovirus.

Bioinformatic analysis indicated that the N-terminal 600-amino-acid fragment of the VD1-ORF4 protein contains three motifs, Exo IDXEExo II (Nx3F/YD) and Exo III (Yx3D), which are conserved in the 3'-5' exonuclease activity domain of prokaryotic and eukaryotic DNA polymerases (Morrison *et al.*, 1991). Moreover, the fragment 706-1004 of VD1-ORF4 protein contains five motifs, Dx2SLYP (motif I), Kx3NSxYG (motif II), Tx2GxAR (motif III), YxDTDS (motif IV) and KxY (motif V), which are conserved in protein-primed DNA polymerase belonging to the family of B DNA polmerase. Protein-primed DNA polymerase initiate DNA replication by using a protein as a primer (Kapitonov *et al.*, 2006). These polymerases display both 3'-5' exonucleolytic and 5'-3' synthetic activities defined by two structurally independent N- and C-terminal domains. The five conserved motifs are involved in synthetic activities of polymerase, such as dNTP binding, DNA synthesis and phosphorylation hydrolysis (Bernad *et al.*, 1990). Taken together, we propose that the protein encoded by *VD1-ORF4* is very likely to be a novel DNA polymerase.

However, there is no experimental evidence showing whether the full length VD1-ORF4 protein is really expressed in insect cells infected with *Bm*PLV-Z. In this study, we constructed a recombinant baculovirus to express the full length protein encoded by the *VD1-ORF4* gene (3318 bp) in Sf-9 cells for characterizing the stability of the full length VD1-ORF4 protein. A 2163-bp fragment of *Bm*PLV-Z *VD1-ORF4* was cloned into pET-30a and expressed in Rosetta 2 (DE3) pLysS. The amino acid sequence encoded by this fragment contains two exonuclease and five polymerase motifs of the hypothetical DNA polymerase. The 6xHis-tagged fusion protein was confirmed by Western blot and mass spectrographic analysis. The fusion protein expressed in *E. coli* was purified by a Ni^2+^-NTA column (Novagen) and then used to raise an antiserum in rabbits according to the method of Sambrook *et al.* (1989). This antiserum was specially employed to detect the VD1-ORF4 protein expressed in Sf-9 cells.

## Materials and Methods

###  Cells and virus

Sf-9 cell, TG1 and *Escherichia coli* Rosetta 2 (DE3) pLysS strains, besides pFasBacHTb and DH10Bac containing the Ac*M*NPV bacmid, were maintained in our laboratory. The Ac*M*NPV BV stocks were prepared by transfecting Sf-9 cells with extracted recombinant bacmids. TC-100 insect medium was supplemented with 10% (v/v) fetal bovine serum (Gibco, USA). Sf-900 II SFM and EXPRES-FIVE SFM were purchased from Invitrogen Company (USA). Restriction enzyme, T4 DNA ligase, and PCR reagents were purchased from TaKaRa Company (Dalian, China). Primers and other reagents were obtained from Shanghai Sangon Bio-technology Corporation (Shanghai, China).

###  Cloning of BmPLV-Z *VD1-ORF4*

The primer pair P1: 5'-CCGGAATTCATGTTTTT AACTGATTTATATAG-3' and P2: 5'-CCGCTCGAGTT ATTCAATTACAACATCATC-3' was designed to amplify the 2163-bp fragment at the 3'-terminal of *Bm*PLV-Z *VD1-ORF4*. The PCR product was cloned into pMD18-T which generated recombinant plasmid pMD18-T-*VD1-ORF4* (2163 bp), and was validated by restriction analysis and DNA sequencing.

The primer pair P3: 5'-CCGGAATTCAAATGCC TTTAGTGAAGATTAC-3' and P2: 5'-CCGCTCGAGTT ATTCAATTACAACATCATC-3' was designed to amplify the 3318-bp full length coding region. The PCR product was cloned into pMD18-T which, in turn, generated plasmid pMD18-T-*VD1-ORF4* (3318 bp), and was subsequently validated by restriction analysis and complete sequencing.

###  Expression of BmPLV-Z VD1-ORF4 (2163 bp) in *E.coli* and Western blot analysis

The 2163-bp fragment was excised from pMD18-T-*VD1-ORF4* (2163 bp) by *Eco*RI and *Xho*I, and subsequently subcloned into the pET-30a expression vector (Novagen, USA) in frame with a 6xHis tag at the N terminus. The recombinant plasmid, pET-30a-*VD1-ORF4* /2163, was verified by restriction analysis and DNA sequencing. RosettaTM 2 (DE3) pLysS cells harboring pET-30a-VD1-ORF4/2163 were grown to an OD600 of 0.6, and then induced by the addition of isopropyl-β-D-thiogalactopyranoside (IPTG) at concentrations ranging from 0.2 to 0.8 mmol/L. After incubation for 10 h at 28 °C, cells were harvested by centrifugation at 7000 g for 15 min at 4 °C. The fusion protein present in the cells was separated in 10% SDS-polyacrylamide gels and stained with Coomassie brilliant blue. Western blot analysis, using anti-6xHis tag antibodies, was applied for confirming the presence of 6xHis tagged fusion proteins. Briefly, this involved resuspending harvest-cell pellets in a SDS-PAGE loading buffer, and their boiling for 10 min, with subsequent analysis. Anti-6xHis tag antibodies were used at a dilution of 1:500. Horseradish peroxidase conjugated goat anti-rat IgG was used as secondary antibody. The immunoreactive proteins were visualized by using DAB staining.

###  Verifying fusion protein expression by way of mass spectrum analysis

The proteins were excised and digested with trypsin, and then analyzed by matrix-assisted laser desorption ionization-time-of-flight mass spectrometry MALDI-TOF (Bruker Daltonics, Germany). Peptide mass information on tryptic peptides from fusion proteins was obtained, and subsequently used to search corresponding proteins in the NCBI database via the MASCOT program.

###  Preparation of an antibody against protein fragment VD1-ORF4

The expressed fusion protein was purified by means of a Ni^2+^-NTA column (Novagen) and used to raise a polyclonal antibody in New Zealand White rabbit. The rabbit was subcutaneously immunized at four different sites by injecting 200 μ g of antigen, with complete Freund's adjuvant. Three weeks later, the rabbit was injected with 200 μ g protein with Freund's incomplete adjuvant as booster injections. Subsequently, two additional booster doses were administered at 3-week intervals. Two weeks after the final dose, serum was separated from blood collected from the rabbit's ear vein, and then stored in 0.1% sodium azide at -20 °C. The anti-VD1-ORF4 antibodies were purified using protein-A-sepharose CL-4B (Sigma, USA), whereupon they underwent SDS-PAGE analysis to check their purity.

###  Expression of *VD1-ORF4* (3318 bp) in Sf-9 cells and Western blot analysis

A Bac-to-Bac baculovirus expression system was used for expressing *Bm*PLV-Z full length *VD1-ORF4*. Briefly, *VD1-ORF4* fragments were excised from pMD18-T-*VD1-ORF4* with *Eco*RI and *Xho*I, and subcloned into pFasBacHTb to generate pFB-*VD1-ORF4*. DH10B cells harboring the Ac*M*NPV bacmid (Ac-bacmid) and helper vector encoding a transposase were transformed with donor plasmid pFB-*VD1-ORF4*, so as to generate recombinant Ac-bacmid-*VD1-ORF4* by transposition. *E.coli* colonies containing recombinant Ac-bacmid-*VD1-ORF4* were screened by blue/white selection according to manufacturer's instructions. Sf-9 cells were transfected with either extracted recombinant Ac-bacmid-*VD1-ORF4* or wild Ac-bacmid, and the resultant supernatants of both transfections harvested.

A monolayer of Sf-9 cells was infected with a mock or recombinant virus supernatant (multiplicity of the infectious virus was 5), in order to obtain expression of 6xHis-VD1-ORF4 protein. Cells were harvested at 72 h post-infection. These were first pelleted and resuspended in phosphate-buffered saline (PBS, pH 7.4), then lysed in a SDS – PAGE loading buffer, and finally analyzed in Western blots. VD1-ORF4 protein specific antibodies were used at a dilution of 1:1000. Horseradish peroxidase conjugated goat anti-rabbit IgG was used as secondary antibody. Visualization of immunoreactive proteins was through DAB staining.

## Results

###  Identification of the target fragment amplified from *Bm*PLV-Z *VD1-ORF4*

**Figure 1:**
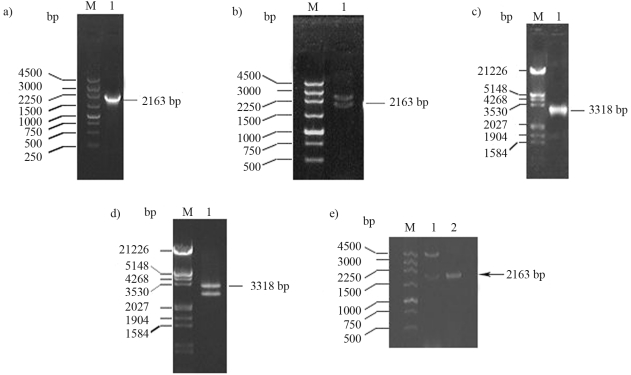
Agarose gel electrophoresis of PCR products and recombinant plasmids digested with *Eco*RI and *Xho*I. (a) Lane M, DNA marker; Lane 1, the 2163-bp fragment product from *VD1-ORF4*. (b) Lane M, DNA marker; Lane 1, pMD18-T-*VD1-ORF4*/2163 digested with *Eco*RI and *Xho*I. (c) Lane M, DNA marker; Lane 1, the 3318-bp fragment product from *VD1-ORF4*. (d) Lane M, DNA marker; Lane 1, pMD18-T-*VD1-ORF4* digested with *Eco*RI and *Xho*I. (e) Lane M, DNA maker; Lane 1, pET-30a-*VD1-ORF4*/2163 digested with *Eco*RI and *Xho*I; Lane 2, the 2163-bp PCR product from *VD1-ORF4*.

A 2163-bp fragment was amplified from *Bm*PLV-Z *VD1-ORF4* by the specific primer pair P1 and P2 (Figure 1a). The PCR products obtained were cloned into pMD18-T (Figure 1b) and then sequenced. In addition, the full length of *VD1-ORF4* was amplified by the specific primer pair P3 and P2 (Figure 1c). The PCR products obtained were cloned into pMD18-T (Figure 1d) and sequenced. The size of obtained sequences corresponded with the expected fragments amplified from *VD1-ORF4*.

###  Construction of the expression plasmid pET-30a-VD1-ORF4/2163

The plasmid pMD18-T-*VD1-ORF4*/2163 was digested with *Eco*RI and *Xho*I, whereupon the released 2163-bp fragment was purified and ligated with pET-30a, which itself was also digested with the same two enzymes, to generate pET-30a-*VD1-ORF4*/2163 (Figure 1e).

###  Expression of fusion protein in *E. coli* and analysis by Western blot and MS

**Figure 2:**
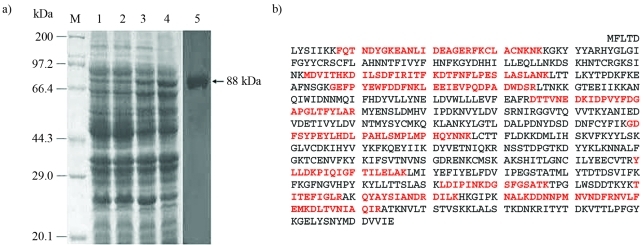
Identification of induced target-protein expression in *E. coli* Rosetta TM 2 (DE3) pLysS, together with Western blot and MALDI-TOF analysis of the induced protein. (a) SDS-PAGE and Western blot analysis of the target protein induced in RosettaTM 2 (DE3) pLysS. Lane M, protein marker; Lane 1, RosettaTM 2 (DE3) plysS with pET-30a-*VD1-ORF4*/2163 induced without IPTG; Lane 2-4, Rosetta TM 2 (DE3) plysS with pET-30a-*VD1-ORF4*/2163 induced by IPTG concentrations of 0.2, 0.5, 0.8 mmol/L, respectively; Lane 5, the target protein was examined with an antibody against the 6xHis tag. (b) Peptide sequences identified by mass spectrometry. The *Bm*PLV-Z *VD1-ORF4* deduced amino acid sequence is shown, and the matched peptide sequences are indicated as red characters.

SDS-PAGE analysis indicated that target protein expression could be induced by different concentrations of IPTG (Figure 2a). In its absence, expression proved to be basal (Figure 2a Lane 1), whereas it was highest with the addition of 0.8 mmol/L (Figure 2a Lane 4). Protein induction was confirmed by Western blot analysis using anti-6xHis tag antibody (Figure 2a Lane 5). The fusion-protein band was excised for MS analysis. The Mascot search was carried out with carbamidomethyl as the fixed modification of cysteine and variable N-terminal Gln-pyroGlu. The fusion protein was identified as part of *Bm*PLV-Z *VD1-ORF4* based on deduced amino acid sequence (Figure 2b).

**Preparation of Specific antibody against *VD1-ORF4***

**Figure 3:**
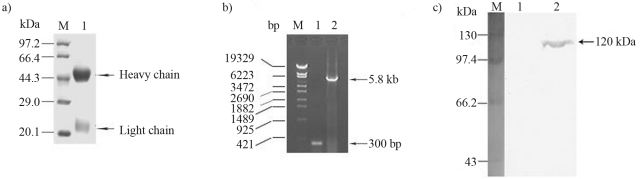
SDS-PAGE analysis of the purified antibody against VD1-ORF4, electrophoretic analysis of Ac-bacmid-*VD1-ORF4* after PCR with pUC/M13 primers, and Western blot analysis to detect *VD1-ORF4* expression in Sf-9 cells. (a) Lane M, protein marker; Lane 1, purified polyclonal antibody. The two chains of purified antibody are respectively indicated by an arrow on the right. (b) Lane M, DNA marker; Lane 1, PCR product amplified from wild Ac-bacmid; Lane 2, PCR product amplified from recombinant Ac-bacmid-*VD1-ORF4*. (c) Lane M, pre-staining protein marker; Lane 1, a protein sample from Sf-9 cells infected with the wild virus was used as control; Lane 2, the target protein was examined with VD1-ORF4 protein specific antibody.

After confirmation of the 6xHis fusion protein, it was purified and subsequently used to immunize rabbits to raise a specific antibody. The harvested antiserum was further purified by using protein-A-sepharose CL-4B, with posterior SDS-PAGE analysis to check the purity of the final product (Figure 3a).

### *VD1-ORF4* expression in Sf-9 cells

The recombinant Ac-bacmid-*VD1-ORF4* was confirmed by PCR, using pUC/M13 primers (Figure 3b). A specific band about 5.8 kb from recombinant Ac-bacmid-*VD1-ORF4* (Figure 3b Lane 2) was amplified, but only a fragment of 300 bp in the empty Ac-bacmid (Figure 3b Lane 1). 6xHis fusion protein expression in Sf-9 cells infected with the recombinant virus was confirmed by Western Blot analysis, using VD1-ORF4 protein-specific antibodies (Figure 3c). Apparently, only a specific band of about 120 kDa was detected in extracts from recombinant virus infected cells (Figure 3c Lane 2), whereas no similar band was detected in extracts from wild virus infected cells (Figure 3c Lane 1), thereby indicating successful expression of the entire *VD1-ORF4* of *Bm*PLV-Z and full length stability of the protein in Sf-9 cells.

## Discussion

*Bm*PLV-Z is a viral agent that causes flacherie disease in silkworms, thus adverse to sericulture. The *Bm*PLV-Z genome has been sequenced by Wang *et al.* (2007). In order to explore the interactions between *Bm*PLV-Z and *Bombyx mori*, there is the urgent need to study the functions of viral genes, especially the *VD1-ORF4* gene encoding a putative DNA polymerase.

There is a significant difference in genome structure between *Bm*PLV-Z and parvoviruses. It is generally accepted that vertebrate parvovirus genomes contain two major blocks of open-reading frames in the same strand, the left-hand region encoding non-structural proteins and the right structural proteins (Srivastava *et al.*, 1983; Cotmore and Tattersall, 1986, 1987; Alexandersen *et al.*, 1988). However, the *Bm*PLV-Z genome consists of two linear single-strand molecules, VD1 and VD2. The sequence of VD1 is quite different from that of VD2, although they share a common terminal sequence of 53 nt at their 5'-ends, which facilitates their forming panhandles. Moreover, there is high homology between the deduced amino acid sequence of *VD1-ORF4* and various protein-primed DNA polymerases (Kapitonov and Jurka, 2006). It has been stated that replication in Bombyx mori parvo-like viruses (Yamanashi isolate) and adenoviruses is alike (Tijssen and Bergoin, 1995). Accordingly, it is very likely that the protein encoded by *VD1-ORF4* is directly involved in virus replication. However, so far, very little is known about the exact replication mechanism of *Bm*PLV-Z. To further investigate this mechanism, it is essential to characterize the protein encoded by *Bm*PLV-Z *VD1-ORF4*.

In this study, a 2163-bp fragment was amplified from *Bm*PLV-Z *VD1-ORF4* and expressed in *E.coli* Rosetta TM 2 (DE3) pLysS. SDS-PAGE analysis revealed the optimal induction conditions to be 0.8 mmol/L of IPTG for 10 h. Nevertheless, basal expression of the desired fragment also occurred in non-induced cells, thus possibly severely affecting host-cell growth, thereby leading to relatively low expression. Originally, *E.coli* BL21 (DE3) was used as the host expression strain in attempt to detect the target protein, but without success. Furthermore, analysis of rare codon distribution (data not shown) indicated that BL21 (DE3) was unsuitable for target protein expression, which suggested the importance of the choice of host bacteria. The recombinant fusion protein was purified on a Ni^2+^-NTA column and used to raise an antiserum in rabbits. The harvested antiserum was purified and used for further research.

Recombinant baculoviruses are widely used to express heterologous genes in cultured insect cells and insect larvae. The baculovirus expression vector system (BEVS) is particularly advantageous for large scale application (Hu, 2005; Kost *et al.*, 2005). Furthermore, the host of *Bm*PLV-Z is an insect. Thus, studies of *Bm*PLV-Z *VD1-ORF4* expression and its function in Sf-9 cells are essential for knowing the physiological role of *Bm*PLV-Z *VD1-ORF4* in infection. Full length *Bm*PLV-Z *VD1-ORF4* expression in Sf-9 cells was brought about by using a recombinant baculovirus. A specific band of about 120 kDa in length was detected in Sf-9 cells infected with recombinant baculovirus, whereas no smaller-sized bands were observed through Western blot analysis, thus indicating that the full length of VD1-ORF4 protein in these cells was stable. However, as putative DNA polymerase might only be involved in *Bm*PLV-Z replication, further studies of its biochemical function in the infection process are called for.

When starting, production of the target protein was relatively meager, through being only visible by the detection of a specific antiserum (data not shown). Many trial tests were undertaken in order to discover the optimal conditions for reaching a higher level of target protein expression. Thus, a certain number of commercially-available culture media, such as Sf-900 II SFM and EXPRES-FIVE SFM, were adapted for Sf-9 cell growth. Surprisingly, the expression level of the target protein was increased after replacing TC-100 by Sf-900 II SFM, thus laying a solid foundation for its purification for functional and structural studies.

The results obtained in this work, together with previous findings, will be very useful in subsequent studies, such as characterization of the VD1-ORF4 protein and the *in vivo* identification of its interacting proteins, thereby constituting a step forward in the precise elucidation of the molecular mechanism of VD1-ORF4 gene action in the life cycle of BmPLV-Z.
